# Genetic nurture versus genetic transmission of risk for ADHD traits in the Norwegian Mother, Father and Child Cohort Study

**DOI:** 10.1038/s41380-022-01863-6

**Published:** 2022-11-16

**Authors:** Jean-Baptiste Pingault, Wikus Barkhuizen, Biyao Wang, Laurie J. Hannigan, Espen Moen Eilertsen, Elizabeth Corfield, Ole A. Andreassen, Helga Ask, Martin Tesli, Ragna Bugge Askeland, George Davey Smith, Camilla Stoltenberg, Neil M. Davies, Ted Reichborn-Kjennerud, Eivind Ystrom, Alexandra Havdahl

**Affiliations:** 1 Division of Psychology and Language Sciences, University College London, London, United Kingdom; 2 Social, Genetic and Developmental Psychiatry Centre, Institute of Psychiatry, King’s College, London, United Kingdom; 3 Nic Waals Institute, Lovisenberg Diaconal Hospital, Oslo, Norway; 4 Department of Mental Disorders, Norwegian Institute of Public Health, Oslo, Norway; 5 MRC Integrative Epidemiology Unit (IEU), University of Bristol, Bristol, United Kingdom; 6 Population Health Sciences, Bristol Medical School, University of Bristol, Bristol, United Kingdom; 7 PROMENTA Research Center, Department of Psychology, University of Oslo, Oslo, Norway; 8 Centre for Fertility and Health, Norwegian Institute of Public Health, Oslo, Norway; 9 NORMENT Centre, Institute of Clinical Medicine, University of Oslo and Division of Mental Health and Addiction, Oslo University Hospital, Oslo, Norway; 10 Norwegian Institute of Public Health, Oslo, Norway; 11 University of Bergen, Bergen, Norway; 12 K.G. Jebsen Center for Genetic Epidemiology, Department of Public Health and Nursing, NTNU, Norwegian University of Science and Technology, Trondheim, Norway; 13 Institute of Clinical Medicine, University of Oslo and Division of Mental Health and Addiction, Oslo University Hospital, Oslo, Norway; 14 School of Pharmacy, University of Oslo, Oslo, Norway

**Keywords:** ADHD, Genetics

## Abstract

Identifying mechanisms underlying the intergenerational transmission of risk for attention-deficit/hyperactivity disorder (ADHD) traits can inform interventions and provide insights into the role of parents in shaping their children’s outcomes. We investigated whether genetic transmission and genetic nurture (environmentally mediated effects) underlie associations between polygenic scores indexing parental risk and protective factors and their offspring’s ADHD traits. This birth cohort study included 19,506 genotyped mother-father-offspring trios from the Norwegian Mother, Father and Child Cohort Study. Polygenic scores were calculated for parental factors previously associated with ADHD, including psychopathology, substance use, neuroticism, educational attainment, and cognitive performance. Mothers reported on their 8-year-old children’s ADHD traits (*n* = 9,454 children) using the Parent/Teacher Rating Scale for Disruptive Behaviour Disorders. We found that associations between ADHD maternal and paternal polygenic scores and child ADHD traits decreased significantly when adjusting for the child polygenic score (*p*_*Δβ*_ = 9.95 × 10^−17^ for maternal and *p*_*Δβ*_ = 1.48 × 10^−14^ for paternal estimates), suggesting genetic transmission of ADHD risk. Similar patterns suggesting genetic transmission of risk were observed for smoking, educational attainment, and cognition. The maternal polygenic score for neuroticism remained associated with children’s ADHD ratings even after adjusting for the child polygenic score, indicating genetic nurture. There was no robust evidence of genetic nurture for other parental factors. Our findings indicate that the intergenerational transmission of risk for ADHD traits is largely explained by the transmission of genetic variants from parents to offspring rather than by genetic nurture. Observational associations between parental factors and childhood ADHD outcomes should not be interpreted as evidence for predominantly environmentally mediated effects.

## Introduction

Intergenerational psychiatry aims to understand how parents contribute to the emergence of psychiatric risk in their offspring [1]. A fundamental question is whether parents affect their offspring via genetic or environmental pathways, or both. Different mechanisms have different implications for intervention strategies aiming to disrupt the cycle of transmission across generations. Here, we implement a novel design capitalising on genomic data from parents and their children to systematically investigate the intergenerational transmission of inattention and hyperactivity-impulsivity traits comprising attention-deficit/hyperactivity disorder (ADHD) [2].

Observational studies have identified many parental factors associated with offspring ADHD: parental psychiatric conditions including ADHD itself, depression, anxiety disorders, schizophrenia, bipolar disorder and autism spectrum disorder (autism) [3–10]; personality traits like neuroticism [11]; cognitive disability [9], lower education and socioeconomic position [12–15]; and substance use including maternal smoking, alcohol consumption and cannabis use during pregnancy [16–20]. Like most complex phenotypes, these parental factors as well as offspring ADHD are partially heritable [21–23], which leaves findings from such studies vulnerable to confounding by shared genetic factors. Genetic variants transmitted from parents to children can independently affect both parental factors and their offspring’s outcomes. This can lead to associations between parental factors and ADHD traits in the absence of environmental pathways of causation [24]. Addressing this issue is fundamental to assess whether modifiable parental factors are likely to be effective targets for intervention.

Several genetically informed designs for causal inference [24, 25] such as family designs, have demonstrated that genetic transmission from parents to their children need to be accounted for in aetiological studies of childhood ADHD [10, 15, 26–28]. However, these studies require specific samples (e.g., twins or adopted children and their parents), leading to both scarcity and paucity of suitable samples. Furthermore, most studies focus on one or a few parental factors at a time. Each design also has its own limitations. For example, in the adoption design, the association between ADHD traits in the adoptive mother and child may be partly due to reverse causation. As such, triangulation of findings between studies that employ genetically informed designs with different underlying assumptions and limitations is essential [29, 30].

**Fig. 1:**
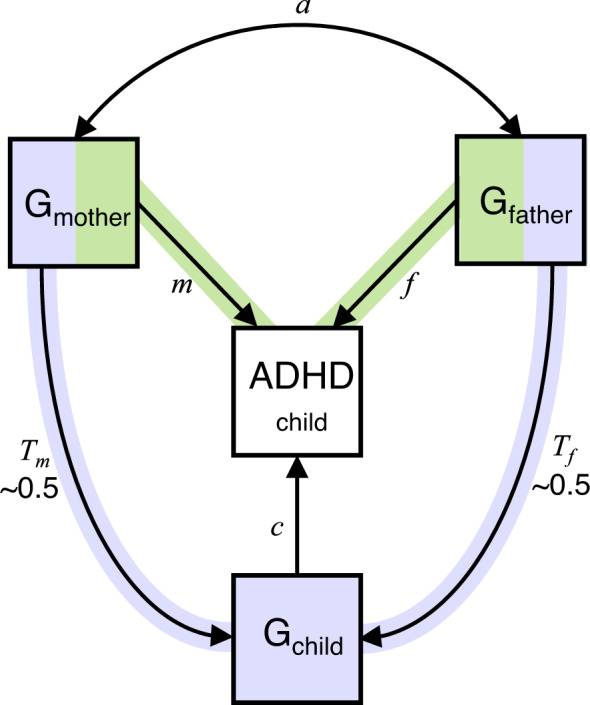
Within-family trio model to investigate the intergenerational transmission of risk. Path diagram illustrating a regression model with polygenic scores for mother (G_mother_), father (G_father_) and child (G_child_) predicting child ADHD traits. Genetic influences shared between the child and their mother (path *T*_*m*_) and father (path *T*_*f*_) are illustrated in purple. Coefficients for path *c* represent direct genetic effects of the child’s polygenic score on their ADHD traits after controlling for the influences of parental polygenic scores. Genetic transmission is the path from parental genetics to child traits via child genetics (e.g., G_mother_ to child ADHD via paths *T*_*m*_ and *c*). Attenuation of associations between parental polygenic scores and child ADHD when adjusting for child polygenic scores is evidence for genetic transmission. Genetic nurture effects (highlighted in green) on childhood ADHD are estimated from path coefficients *m* for mothers and *f* for fathers. Coefficients for genetic nurture paths *m* and *f* reflects the effects of parental genotypes on children’s traits after statistically controlling for the genetic influences transmitted from parents to their child and for covariation between parental polygenic scores (path *a*). Additional influences captured by genetic nurture effect estimates can also include familial environments such as sibling or grandparent effects, and estimates may be influenced by demographic factors such as population stratification and assortative mating. Note that path *a* will be near zero in the absence of assortative mating, which was the case for correlations between most parental polygenic scores used in the analyses, apart from educational attainment (*r* = 0.11; Supplementary Table 3).

Intergenerational transmission can be investigated in population-based samples with genotype data on parents and offspring and phenotypic measures on offspring outcomes [31, 32]. Instead of phenotypic parental measures, genetic variants associated with the parental factor (e.g., ADHD) can be combined into a polygenic score which reflects an individual’s genetic propensity. As illustrated in Fig. 1, relevant estimates can be obtained by regressing children’s outcomes jointly onto their own and both their parents’ polygenic scores. This multivariate trio model provides estimates of: (i) the direct genetic effects on the offspring’s outcome due to parent-to-offspring allele transmission (path *c*), and (ii) the indirect genetic effects of parental alleles not due to influences of alleles inherited by the offspring (paths *m* and *f*). In this trio design, the associations between parental polygenic scores and offspring traits are adjusted for the offspring polygenic score to account for the confounding induced by the transmission of genetic variants from parents to offspring. Such adjusted associations are called indirect genetic effects, which originate in parental genomes but affect their offspring via environmental pathways (i.e., independently of genetic transmission). In the case of ADHD, parental ADHD variants influence parental ADHD traits, which in turn may shape the child rearing environment (i.e., nurture), ultimately affecting their offspring’s ADHD traits. This indirect parental genetic effect is henceforth referred to as “genetic nurture” [32]. Importantly, all genetic variants comprising parental genomes can contribute to the genetic nurture effect, for example by influencing parenting behaviours, whether or not variants are transmitted to the offspring. In contrast, only transmitted parental alleles can be involved in direct genetic effects in the offspring generation. In the trio design, direct genetic effects are estimated from the association between children’s polygenic scores and their ADHD traits whilst adjusting for the maternal and paternal polygenic scores. In this way, confounding by genetic nurture effects is removed. This approach is approximately analogous to computing distinct polygenic scores based on transmitted and non-transmitted alleles, and using these to derive estimates of genetic nurture and direct genetic effects [32]. These two approaches have been shown to produce similar results [33].

To date, only one study, in the Netherlands Twin Register cohort, has used polygenic scores to investigate the association between parental factors and their offspring’s ADHD traits [34]. They found that the association between parental factors and their offspring’s ADHD traits could mainly be attributed to genetic transmission rather than to genetic nurture. However, the Netherlands Twin Register study focused on only two parental factors (ADHD and education) and had a relatively small sample (*N* = 2518 families).

In the present study, we use a large population-based cohort of genotyped mother-father-offspring trios to systematically investigate a range of putative parental risk factors for offspring ADHD, including psychiatric conditions, substance use, neuroticism, educational attainment (EA) and cognitive performance. We jointly model maternal, paternal and offspring polygenic scores to quantify genetic versus environmental routes of risk transmission.

## Samples and methods

### Participants and genotype data

The Norwegian Mother, Father and Child Cohort Study (MoBa) [35, 36] is a population-based pregnancy cohort study conducted by the Norwegian Institute of Public Health. Participants were recruited from across Norway during 1999–2008. Mothers consented to participation in 41% of the pregnancies. The cohort includes 114,500 children, 95,200 mothers and 75,200 fathers. Blood samples were obtained from both parents during pregnancy and from mothers and children (umbilical cord) at birth. The Medical Birth Registry (MBRN) is a national health registry containing information about all births in Norway and was used to obtain children’s year of birth and sex. The current study is based on version 12 of the quality-assured data files released for research in January 2019. The establishment of MoBa and initial data collection was based on a license from the Norwegian Data Protection Agency and approval from The Regional Committees for Medical and Health Research Ethics (REK). MoBa is based on regulations based on the Norwegian Health Registry Act. The current study was approved by REK (2016/1702).

The eligible sample was MoBa families with genotypic data [37] available for mother-father-offspring trios. After quality control (procedures are outlined in the Supplementary Note), we excluded up to second-degree relatives within generations from within groups of related family trios (leaving no families with e.g., siblings in the parental or offspring generation) whilst prioritising trios with complete phenotypic data, resulting in 19,506 trios with genotypic data available (Supplementary Fig. 1).

### Measures

#### Childhood traits of attention-deficit hyperactivity disorder

Children’s ADHD traits were reported by mothers using the Parent/Teacher Rating Scale for Disruptive Behaviour Disorders (RS-DBD) [38] that contains 18 items related to DSM-IV criteria for ADHD. Items were rated on a four-point scale. Participants with fewer than half completed items were excluded, which resulted in 9,454 families with phenotypic data available. We computed a total ADHD summary score and repeated analyses with inattention and hyperactivity-impulsivity subscales (9 items each). To mitigate bias due to attrition in cohort studies, multiple imputation was performed to derive ADHD scores for the full study sample (*N* = 19,506 genotyped family trios) imputed from polygenic scores (which were available for all eligible participants and, thus, not imputed) and 23 auxiliary variables previously found to predict ADHD in MoBa [39], including records of ADHD diagnoses and earlier measures of ADHD traits, socioeconomic variables and child motor and language development (see Supplementary Note for additional information). Descriptive statistics on auxiliary variables and evaluation of imputation quality are provided in Supplementary Table 1 and Supplementary Figs. 2-3.

#### Polygenic scores indexing parental factors

Polygenic scores were used as indicators of genetic liabilities for the corresponding parental factors (e.g., the maternal polygenic score for depression). Polygenic scores are individual-level scores that summarize common variant genetic risk for a given phenotype. The scores are calculated as the weighted sum of effect alleles for all measured single nucleotide polymorphisms (SNPs) in an individual. SNP weights are obtained from summary statistics from Genome-Wide Associations Studies (GWAS; e.g. for depression).

We derived polygenic scores corresponding to 12 putative parental risk factors identified based on previous literature [40–51] and the availability of well-powered GWAS of relevant phenotypes (Supplementary Table 2). One polygenic score per parental factor was generated for each mother, father and child using PRS-PC [52]. PRS-PC takes the first principal component of polygenic scores computed at several *p*-value thresholds, which increases the predictive accuracy of polygenic scores while avoiding overfitting (see Supplementary Note).

Correlations between parental polygenic scores are provided in Supplementary Table 3. Compared to family trios with complete data, families with missing offspring ADHD scores had higher polygenic scores for ADHD, schizophrenia, depression, neuroticism and smoking, and lower polygenic scores for cognition and EA (Supplementary Table 4).

### Data analysis

Prior to analyses, polygenic scores were standardized and residualized for genotyping and imputation batch, array effects, and the 10 first principal components to control for population stratification.

#### Unadjusted models

Linear regressions of offspring ADHD traits on each polygenic score were conducted. False Discovery Rate (FDR) of 5% was applied to account for multiple testing [53], the number of tests being equal to 36, i.e. 12 parental factors times three family members.

#### Trio models

For each of the 12 parental factors, offspring ADHD traits were regressed onto three polygenic scores, i.e., for father, mother, and child (Fig. 1). FDR was applied as above.

#### Complementary analyses

We considered parental factors for which at least one family member’s polygenic score significantly predicted offspring ADHD traits in the trio models, jointly in one model across parental factors. This was to assess whether correlations between polygenic scores could affect estimates of genetic nurture and genetic transmission. Unadjusted and trio models were also conducted on the sample with complete phenotypic data available (prior to multiple imputation for missingness), as well as on the two ADHD subscales (inattention and hyperactivity-impulsivity).

All analyses controlled for child sex and year of birth. We tested whether estimates from the unadjusted, trio and across factor models differed significantly across models (*p*_*Δβ*_ < 0.05) [54].

## Results

**Fig. 2:**
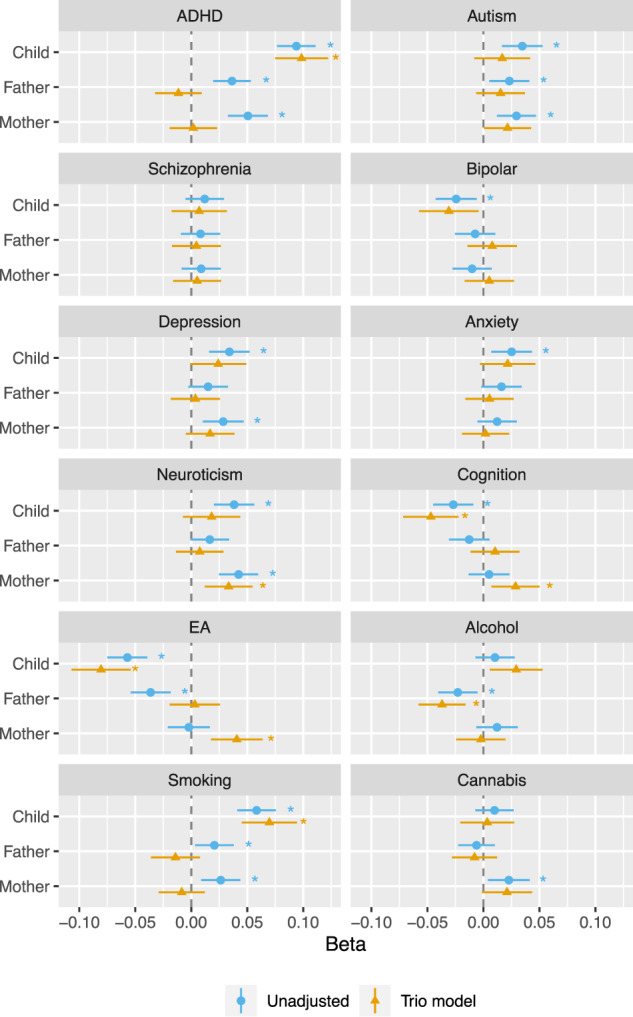
Family trios’ polygenic scores predicting child ADHD traits before and after adjusting for polygenic scores of other family members (within risk factors). ADHD Attention-deficit/hyperactivity disorder, Autism Autism spectrum disorder, EA Educational attainment. ADHD scores were standardized to have a mean of zero and a standard deviation of 1. Trio results adjusted for polygenic scores of other family members. All models adjusted for child sex and year of birth. *N* = 19,506 family trios. * indicates estimates that remained significant after correction for multiple testing (36 tests) at a false-discovery rate (FDR) of 5%.

Sample demographic characteristics are summarized in Table 1. Results from unadjusted and trio models are shown in Fig. 2 and Supplementary Table 5.

**Table 1:** Descriptive statistics for child ADHD scores (age 8 years) and sample demographic characteristics.

		*N*	Mean (*SD*)	Median	Range
**ADHD total score**	*Female*	4570	25.25 (6.43)	24	18–70
	*Male*	4884	27.75 (7.96)	26	18–72
**Inattention subscale**	*Female*	4570	13.23 (3.68)	12	9–36
	*Male*	4884	14.65 (4.47)	14	9–36
**Hyperactivity-impulsivity subscale**	*Female*	4570	12.01 (3.52)	11	9–36
	*Male*	4884	13.10 (4.32)	12	9–36
**Parental age at baseline**	*Mothers*	19,506	30.08 (4.46)	30	17–47
	*Fathers*	19,506	32.51 (5.15)	32	18–61
			**Mothers**		**Fathers**
			***n*** **(%)**		***n*** **(%)**
**Highest qualification**	Lower secondary school		378 (2.07)		675 (3.84)
	Upper secondary school, basic		795 (4.36)		906 (5.15)
	Vocational upper secondary school		2280 (12.51)		4469 (25.42)
	Upper secondary school, completed		2593 (14.23)		2158 (12.27)
	Higher education, undergraduate level		7761 (42.59)		5087 (28.93)
	Higher education, graduate level		4414 (24.22)		4286 (24.38)
**Household income**	No income		318 (1.71)		108 (0.6)
	<150,000 NOK		2629 (14.12)		919 (5.07)
	151,000–199,999 NOK		1922 (10.32)		673 (3.72)
	200,000–299,999 NOK		6369 (34.35)		3795 (20.95)
	300,000–399,999 NOK		5033 (27.03)		6264 (34.58)
	400,000–499,999 NOK		1457 (7.83)		3277 (18.09)
	> 500,000 NOK		863 (4.64)		3076 (16.98)

*ADHD* Attention-deficit/hyperactivity disorder, *SD* standard deviation, *NOK* Norwegian Krone. Sample demographics provided for participants with complete data (prior to multiple imputation for missing ADHD scores).

### Polygenic scores for ADHD

As reported in Fig. 2, the offspring and both parental ADHD polygenic scores predicted offspring ADHD traits in unadjusted models (i.e., not adjusting for polygenic scores of other family members). In trio models, controlling for parental polygenic scores, the effect of offspring polygenic scores was not attenuated and remained significant indicating direct genetic effects on ADHD traits (illustrated as path *c* in Fig. 1). Conversely, parental associations were attenuated compared to estimates from unadjusted models (*p*_*Δβ*_ = 1.48 × 10^−14^ for paternal and *p*_*Δβ*_ = 9.95 × 10^−17^ for maternal estimates), findings which indicate that genetic transmission accounted for associations between parental polygenic scores and offspring ADHD traits, with little evidence for genetic nurture (paths *f* and *m* in Fig. 1).

### Direct genetic effects and genetic transmission

For smoking polygenic scores, Fig. 2 shows a similar pattern of results suggesting direct genetic effects and attenuation of parental polygenic score associations in trio models (*p*_*Δβ*_ = 3.93 × 10^−7^ for paternal and *p*_*Δβ*_ = 2.12 × 10^−10^ for maternal estimates) – findings in line with the genetic transmission of risk. Offspring’s polygenic scores for cognitive performance and EA similarly indicated direct genetic effects. The paternal polygenic score for EA showed evidence of attenuation between unadjusted and trio models (*p*_*Δβ*_ = 1.42 × 10^−8^), consistent with the genetic transmission.

### Genetic nurture

The maternal polygenic score for neuroticism was associated with offspring ADHD traits in unadjusted models and remained significant in trio models, which supports genetic nurture as a mechanism linking maternal neuroticism to offspring ADHD traits.

The findings suggested some genetic nurture effects in unexpected directions, which should be interpreted with caution (see Discussion). The paternal polygenic score for alcohol use was negatively associated with offspring ADHD traits in both unadjusted and trio models, which would suggest genetic nurture as a mechanism linking paternal liability to drink more drinks per week with decreased ADHD traits in offspring. In trio models only, higher maternal polygenic scores for cognitive performance and EA associated with increased offspring ADHD traits.

To further investigate the negative genetic nurture effect of paternal alcohol use reported above, we reran the analyses using polygenic scores for alcohol dependence [55, 56]. This additional analysis indicated no direct genetic effect (*β* = 0.01, 95% CI: −0.02 to 0.04), nor genetic nurture effects from either parent (paternal *β* = 0.004, 95% CI: −0.02 to 0.03 and maternal *β* = 0.02, 95% CI: −0.01 to 0.04; Supplementary Table 6).

Some parental polygenic scores were associated with offspring traits in unadjusted models, and while confidence intervals for genetic nurture estimates overlapped with zero, trio model estimates showed little evidence of attenuation (suggesting genetic transmission) for paternal autism (*p*_*Δβ*_ = 0.220) and for maternal autism (*p*_*Δβ*_ = 0.183), depression (*p*_*Δβ*_ = 0.051), and cannabis use (*p*_*Δβ*_ = 0.796). As such, genetic nurture effects may not have been supported due to limited power of current polygenic scores.

### Complementary analyses

Results on the sample prior to multiple imputation of missing phenotypic data (Supplementary Table 7) were consistent with results obtained after imputation. Compared to effect estimates reported in Fig. 2, estimates from the across-parental factor model did not differ significantly in magnitude (Supplementary Table 8).

Results for trio models on offspring inattention and hyperactivity-impulsivity subscales (Supplementary Tables 9, 10) yielded findings similar to those for the total ADHD scores. However, for hyperactivity-impulsivity, the unexpected genetic nurture estimates for paternal alcohol use and for maternal cognition and EA, as well as the direct genetic effects for offspring cognition did not reach statistical significance after correction for multiple testing.

## Discussion

We examined whether genetic transmission or genetic nurture most likely explain the intergenerational transmission of ADHD traits. Specifically, we estimated the associations of polygenic scores indexing genetic liability for putative parental (risk and protective) factors and offspring ADHD traits. We used a multivariate within-family trio design that simultaneously models polygenic scores for parents and offspring. The trio design provides estimates of genetic nurture, unconfounded by direct genetic effects due to parent-to-offspring genetic transmission. Conversely, it provides estimates of direct genetic effects on offspring ADHD traits accounting for confounding by genetic nurture effects. Several parental polygenic scores correlated with offspring ADHD traits prior to accounting for genetic transmission, including ADHD itself, autism and smoking, maternal scores for depression, neuroticism and cannabis use, and paternal scores for EA and alcohol use. Trio models provided strong evidence that the offspring polygenic scores for ADHD and smoking predicted ADHD traits while parental effects were no longer evident and attenuated compared to the unadjusted parental effects. These findings support the role of parent-to-child genetic transmission of ADHD, smoking and EA-associated alleles as important mechanisms underlying associations between these parental factors and their children’s ADHD traits, with little evidence of genetic nurture. Evidence of genetic nurture was found in the case of maternal neuroticism since higher maternal polygenic liability associated with children’s ADHD traits in trio models.

### Genetic transmission versus genetic nurture

We found that both parental ADHD polygenic scores associated with offspring ADHD traits prior to accounting for genetic transmission. However, that was no longer the case in within-trio analyses and associations between parental polygenic scores and offspring ADHD attenuated significantly, consistent with the genetic transmission. This finding is in line with a previous polygenic score study in a Dutch sample [34]. An adoption study found evidence that ADHD traits in biological mothers predicted child ADHD traits, in line with genetic transmission [26]. The study also found an association of ADHD traits in adoptive mothers and child ADHD, possibly consistent with nurture effects. However, the latter association may arise from reverse causation – e.g., the adoptive mother may find it harder to focus or stay attentive with a hyperactive child. Such reverse causation cannot happen in the design we used since offspring traits cannot change parental genotypes.

Similar patterns were observed for smoking, suggesting that genetic transmission partly explains the association between parental smoking and offspring ADHD traits. These findings are consistent with evidence from multiple genetically informed and causal inference methods, showing that the association between maternal smoking and offspring ADHD traits is likely due to genetic transmission, rather than to the impact of smoking per se [28, 57–59]. Furthermore, our findings show that the offspring polygenic score for smoking is independently associated with ADHD traits in 8-year-old children, i.e. prior to smoking initiation. This confirms that genetic variants implicated in smoking liability are associated with the emergence of ADHD independently of (parental) smoking itself. GWAS for smoking have identified variants implicated in dopaminergic pathways [60], which are also implicated in ADHD [40]. Such variants can contribute to smoking liability in parents, but in children, can also contribute to the emergence of ADHD. This genetic transmission of risk can inflate or generate spurious phenotypic associations between parental smoking and child ADHD.

We found evidence that maternal genetic liability to neuroticism not transmitted to their offspring associated with offspring ADHD traits – findings in line with environmental mechanisms of transmission (e.g., genetic nurture). Neuroticism reported by parents have been phenotypically associated with parenting difficulties [61], which may explain the association we found between maternal genetic variation and offspring traits.

Contrary to previous evidence that children’s ADHD is associated with lower parental cognitive performance and EA [8, 12], we found little evidence that maternal polygenic scores for these factors predicted offspring ADHD traits. After accounting for genetic transmission, we found associations with maternal predisposition to *higher* EA and cognition, a finding which was not robust across complementary analyses. Instead, our findings suggest that direct genetic transmission likely explains the direction of these genetic nurture effects since offspring predisposition to lower EA and cognition remained stronger predictors of their ADHD traits in trio models.

We also found that paternal genetic predisposition to drinking *fewer* drinks per week associated with offspring ADHD traits in both unadjusted and trio models, albeit not robustly so in complementary analyses of the hyperactivity-impulsivity subscale. A possible reason for this direction of association may be that lower polygenic scores for the number of drinks consumed per week index lower socioeconomic status [62]. Nonetheless, our results do not support the notion that more regular parental alcohol use is an environmental risk factor contributing to children’s ADHD despite previous evidence [16, 17, 19, 20]. Furthermore, trio models using polygenic scores for alcohol dependence (instead of quantity of drinks per week) did not support genetic nurture effects.

### Implications for intergenerational psychiatry

Our findings suggest that genetic transmission plays an important role in explaining associations between parental factors and their children’s ADHD traits. Genetic transmission means that phenotypes arise separately in parents and children due to genetic influences they share, but independently of the environment created by parents. Conversely, genetic nurture effects imply that some parental factors matter as they foster environments that affect their offspring (either within the home like parenting or outside like school selection). Implications for interventions are diametrically opposed. In the case of genetic nurture, the effects of interventions targeting parental factors would be expected to affect child attention and hyperactive-impulsive behaviours and cascade across generations. In the case of sole genetic transmission, interventions directly targeting parental factors may successfully improve parental health, but benefits are unlikely to transfer to the child’s attention and hyperactive-impulsive behaviours. Instead, interventions directly aiming to support regulation of attention, activity and impulsivity in children may be more beneficial, whether such interventions are medical, school-based or parent-mediated [63]. Such findings for ADHD traits contrast with findings for EA in children where substantial genetic nurture effects have been found [31, 32]. As such, we should not be expecting universal patterns when it comes to explaining the role of intergenerational risk factors in children’s developmental outcomes. Emerging genetically informed methods [24, 31, 64, 65] should shortly render a detailed depiction of the intergenerational transmission of risk for psychiatric traits.

### Limitations

Despite relying on the largest genotyped cohort of trios so far, power may be an issue to detect small intergenerational effects, especially given the limited accuracy of current polygenic scores. However, power unlikely accounts for our finding that genetic transmission is prominent in explaining the intergenerational transmission of risk for ADHD. For example, maternal genetic nurture effects for ADHD were close to zero with an upper CI of 0.023, whereas the lower CI for child-direct genetic effects was 0.075. Replications of findings in multiple cohorts will help with addressing limitations such as generalizability and power. In the meanwhile, while our findings clearly demonstrate the importance of genetic risk transmission, the limited evidence of genetic nurture effects should be considered as suggestive rather than definitive.

Although polygenic scores can, under strict assumptions, provide estimates of the causal effect of risk factors equal to those obtained from Mendelian randomisation [66], explicit causal inference methods such as intergenerational Mendelian randomization should be implemented in future analyses if genetic nurture effects were to be detected.

While mothers rated their children’s ADHD traits (model outcome), polygenic scores for the child, the father and even the mother (model predictors) did not depend on maternal ratings, limiting possible biases due to shared raters. However, maternal liability to ADHD as indexed by polygenic scores may still influence how they rate their children’s traits. Replication of our findings with non-parental ADHD ratings would be informative. We also did not account for variation in parental involvement in children’s upbringing (e.g., one parent less involved after separation). Future studies should test whether genetic nurture effects are moderated by parental involvement (e.g., larger for the more involved parent).

We cannot rule out possible bias due to assortative mating or population stratification. For educational attainment, maternal and paternal polygenic scores were correlated (*r* = 0.11), which suggests assortative mating (in Fig. 1, path *a* will not be near zero). However, simulation research indicates that assortative mating and population stratification may lead to inflated estimates of genetic nurture while estimates of direct parent-to-offspring genetic transmission effects are expected to be unaffected [33]. As such, our findings of direct genetic effects and limited evidence for genetic nurture should hold robust despite these possible limitations.

In conclusion, our study provides the most reliable evidence from genomic data to date, and the first evidence on many parental factors considered here, that genetic transmission may partly account for associations between some parental factors and children’s ADHD traits. Our findings also corroborate results from other genetically informed designs on the importance of genetics in the intergenerational transmission of ADHD. The implication for psychiatry is that the benefits of interventions targeting parental risk factors (e.g., parental ADHD or smoking) are likely to be restricted to the parental generation rather than extend across generations to the child’s ADHD. Directly targeting children’s traits should be more effective to improve children’s outcomes.

## Supplementary information

Supplementary material

## Data Availability

The study website provides details on how to access data and information on the available variables (https://www.fhi.no/en/studies/moba/for-forskere-artikler/viktige-dokumenter-for-moba-forskere/). GWAS summary statistics used to compute polygenic scores are available from publicly available repositories from GWASatlas (https://atlas.ctglab.nl/) and the Psychiatric Genomics Consortium website (https://www.med.unc.edu/pgc/download-results/).
