# Supplementary material for: Genetic nurture versus genetic transmission of risk for ADHD traits in the Norwegian Mother, Father and Child Cohort Study
Source: Mol Psychiatry. 2022 Nov 16;28(4):1731–8. doi: 10.1038/s41380-022-01863-6 (PMC10208953; doi:10.1038/s41380-022-01863-6)
Supplement: Supplementary file 1 — Supplementary material [file 41380_2022_1863_MOESM1_ESM.pdf]

Supplementary Material

Identifying intergenerational risk factors for ADHD traits using polygenic scores in the Norwegian Mother, Father and Child Cohort

Jean-Baptiste Pingault, Wikus Barkhuizen, Biyao Wang, Laurie J. Hannigan, Espen Moen Eilertsen, Elizabeth Corfield, Ole A. Andreassen, Helga Ask, Martin Tesli, Ragna Bugge Askeland, George Davey Smith, Camilla Stoltenberg, Neil Davies, Ted Reichborn-Kjennerud, Eivind Ystrom, Alexandra Havdahl

Table of contents

MoBa genotype data and quality control 2

Summary statistics used to conduct polygenic scores 2

Polygenic score computation 4

Multiple imputation of ADHD trait scores to account for missingness 4

Supplementary Figure 1. STROBE diagram of eligible MoBa sample. 5

Supplementary Figure 2. Density plot comparing distributions of complete and imputed data. 6

Supplementary Figure 3. Box-and-whisker plot comparing distributions of complete and imputed data. 6

Supplementary Table 1. Variables included during multiple imputation for missingness of ADHD trait scores. . 7

Supplementary Table 2. Genome-wide association study summary statistics used to compute polygenic scores. 8

Supplementary Table 3. Correlations between trio's polygenic scores for each risk factor (*N* = 19,506 families). 9

Supplementary Table 4. Comparison of mean standardized polygenic scores between families with and without child ADHD scores available. 10

Supplementary Table 5. Family trios' polygenic scores predicting child ADHD traits before and after adjusting for polygenic scores of other family members (corresponding to Figure 1). 11

Supplementary Table 6. Alcohol dependence polygenic scores predicting child ADHD traits. 12

Supplementary Table 7. Family trios' polygenic scores predicting child ADHD traits before and after adjusting for polygenic scores of other family members (within risk factors) in sample with complete phenotypic data available (*N* = 9454). 13

Supplementary Table 8. Polygenic scores predicting child ADHD traits adjusted for polygenic scores across risk factors. 14

Supplementary Table 9. Family trios' polygenic scores predicting child inattention traits before and after adjusting for polygenic scores of other family members (within risk factors). 15

Supplementary Table 10. Family trios' polygenic scores predicting child hyperactivity-impulsivity traits before and after adjusting for polygenic scores of other family members (within risk factors). 16

MoBa genotype data and quality control

This study used quality controlled genotypic data from the Norwegian Mother, Father and Child cohort (MoBa). The cohort consisted of approximately 98,000 individuals (approximately 32,000 family trios) and was genotyped in ten batches. Three batches were genotyped at the NTNU Genomics Core Facility (Trondheim, Norway) using the Illumina HumanCoreExome (Illumina, San Diego, USA) genotyping array. The version 12 1.1 chip with 542,585 SNPs was used to genotype the HARVEST 12a batch of 18,972 individuals and the HARVEST 12b batch of 1,692 individuals. Meanwhile, the version 24 1.0 chip with 547,644 SNPs was used to genotype the HARVEST 24 batch that included 12,874 individuals. Two batches were genotyped at ERASMUS MC (Rotterdam, the Netherlands) using the Illumina Global Screening Array (Illumina, San Diego, USA) version 24 1. The ROTTERDAM 1 batch included 17,949 individuals and 692,367 SNPs, while the ROTTERDAM 2 batch included 9,041 individuals and 692,338 SNPs. Five batches were genotyped at deCODE Genetics (Rekjavik, Iceland) using a variety of genotyping Illumina arrays. The Global Screening Array (Illumina, San Diego, USA) version 24 1 array was used to genotype the NORMENT Feb 2018 batch of 9,632 individuals and 693,143 SNPs. The Illumina Human OMNIExpress (Illumina, San Diego, USA) version 24v1.0 was used to genotype the NORMENT Jan 2015 batch of 2,983 individuals and 710,146 SNPs and the NORMENT Jun 2015 batch of 2,983 individuals and 708,882 SNPs. Finally, the Illumina Infinium OMNIExpress (Illumina, San Diego, USA) version 24v1.2 was used to genotype the NORMENT May 2016 batch of 17,608 individuals and 712,628 SNPs and the ADHD/TED batch of 5,410 individuals and 713,599 SNPs.

Quality control of the genotypic data was performed using PLINK 1.9[1] and KING 2.2.4.[2] Imputed dosages at or above 90% certainty were converted into hard genotypes. High quality SNPs were selected by removing SNPs with INFO < 0.8 in all batches, with a MAF < 1% and duplicate SNPs. Participants of European ancestry were identified using principal component analyses conducted on a sample with a SNP call rate threshold of 95%, individuals with a call rate threshold of 95% and a Hardy-Weinberg equilibrium (HWE) threshold of $p < 0.001$.

The core European sample were then filtered by removing SNPs and individuals with high levels of missingness using a call rate threshold for SNPs of 98% and for individuals of 98%. Variants that deviated from HWE were removed at a threshold of $p < 1 \times 10^{-6}$. Individuals who deviated from the samples' heterozygosity rate mean were removed at F +/-0.2. Sex was confirmed by identifying and excluding discrepancies between sex reported in the dataset and sex based on X chromosome heterozygosity. Duplicate individuals were confirmed by running identity-by-descent analysis between pairs of individuals reported to be duplicate. For duplicate individuals, SNPs with a concordance rate of <99% were excluded and one individual from duplicate pairs were removed based on missingness. Relatedness within and across batches were identified in KING[2] (up to second degree relatives between pairs of individuals). Families with more than 5% Mendel error were removed and SNPs with more than 1% Mendel error excluded. Cryptic relatedness was checked based on PCA with and without reference. SNPs with significant associations with genotyping batches were removed. The quality control steps outlined in this paragraph were repeated after removing sex chromosomes.

To avoid overlap between GWAS discovery samples used to compute polygenic scores and the target sample, an additional 198 families were excluded from the analysis since participants from these families were included in the GWAS for schizophrenia and autism.

Summary statistics used to conduct polygenic scores

Polygenic scores were computed for risk factors for which GWAS summary statistics were available from samples of European decent, and from GWAS that were of sufficient power to conduct polygenic score analyses based on established criteria:[3,4] SNP-heritability (SNP-h^2) estimates higher than 0.05 and SNP-h^2 Z-scores (calculated as SNP-h^2 divided by its standard error) higher than 2.

ADHD. Summary statistics were obtained from a genome-wide association study (GWAS) on ADHD[5] from https://www.med.unc.edu/pgc/download-results/. The ADHD GWAS was performed on 19,099 ADHD cases and 34,194 controls consisting of European samples from the Lundbeck Foundation Initiative for Integrative Psychiatric Research (iPSYCH) and the Psychiatric Genomics Consortium (PGC).[5] Cases in iPSYCH were identified based on ICD-10 criteria obtained from psychiatric diagnoses obtained from a national research register. Case definitions for PCG cohorts has been described elsewhere.[6]

Autism Spectrum Disorder (ASD). Summary statistics were downloaded from https://ipsych.dk/en/research/downloads/ and were based on a GWAS meta-analysis of iPSYCH and PGC samples (18,382 cases and 27,969 controls).[7] Cases were based on validated registry-based diagnoses of ASD according to ICD-10 criteria for iPSYCH and for the PGC described elsewhere.[8]

Schizophrenia. The GWAS summary statistics is available at http://walters.psycm.cf.ac.uk/ and was based on a meta-analysis of PGC and CLOZUK samples resulting in 40,675 schizophrenia cases and 64,643 controls.[9] Cases were identified based on DSM-IV criteria for schizophrenia or schizoaffective disorder.

Bipolar disorder. Summary statistics, available from the PGC (https://www.med.unc.edu/pgc/results-and-downloads) was based on a GWAS meta-analysis on 20,352 cases and 31,358 controls. Different clinical interview formats were used to diagnose bipolar disorder, described in full elsewhere.[10]

Depression. Summary statistics for depression were obtained from a meta-GWAS[11] on 170,756 cases and 329,443 controls of European descent (excluding 23andMe participants) obtained from https://atlas.ctglab.nl/traitDB/4293. Depression was defined as either having a diagnosis of Major Depressive Disorder based on clinical interviews, electronic healthcare records, or self-report, and based on self-reported help-seeking or symptoms related to a broad depression phenotype.

Anxiety disorder. GWAS summary statistics of 25,453 anxiety cases and 58,113 controls were downloaded from https://www.kcl.ac.uk/people/kirstin-purves.[12] Participants were those who responded to the online mental health questionnaire in the UK Biobank. Cases were identified based on self-reported lifetime diagnosis of: Anxiety, nerves or generalised anxiety disorder; social anxiety or social phobia; agoraphobia; any other phobia (e.g. disabling fear of heights or spiders); and panic attacks. Additional cases were identified based on CIDI criteria for lifetime generalised anxiety disorder. Cases were excluded if they indicated a lifetime diagnosis of schizophrenia, bipolar disorder, anorexia nervosa, bulimia nervosa, any other type of psychosis or psychotic illness, autism, Asperger's or autistic spectrum disorder, and attention deficit or attention deficit and hyperactivity disorder (ADHD).

Neuroticism. Results from the neuroticism GWAS[13] were downloaded from https://atlas.ctglab.nl/traitDB/3795. The GWAS was performed on 390,278 European participants from the UK Biobank and the Genetics of Personality Consortium (summary statistics did not include 23andMe participants). In the UK Biobank, neuroticism was assessed using 12 items from the Eysenck Personality Questionnaire Revised Short Form (EPQ-RS)[14] and in the Genetics of Personality Consortium with 12 items from the NEO-FFI.[15]

Cognition. The intelligence meta-GWAS[16] was conducted on 269,867 participants of European decent. This meta-analysis included GWAS on phenotypes relating to different domains of cognitive functioning, which were assessed using a range of standard neurocognitive tests. Summary statistics are available from https://ctg.cncr.nl/software/summary_statistics.

Educational attainment. A version of the summary statistics for Educational attainment[17] that did not include MoBa or 23andMe participants was obtained from the authors. Summary statistics were for a meta-analysis of GWAS conducted on samples restricted to participants of European descent and educational attainment was assessed as the number of years of schooling individuals completed.

Alcohol use. Summary statistics for alcohol use[18] were downloaded from https://atlas.ctglab.nl/traitDB/4069. The GWAS was conducted on 414,343 UK Biobank participants of European descent (excluding 23andMe samples). Alcohol use was coded as the average number of alcoholic drinks consumed per week.

Smoking. Smoking was a continuous measure coded based on a lifetime smoking index[19] devised to capture the heaviness and duration of tobacco smoking and included non-smokers (N = 462,690). GWAS summary statistics[20] were obtained from https://data.bris.ac.uk/data/dataset/10i96zb8gm0j81yz0q6ztei23d.

Cannabis use. Summary statistics for a meta-GWAS of lifetime cannabis use[21] was downloaded from https://www.ru.nl/bsi/research/group-pages-0/substance-use-addiction-food-saf/vm-saf/genetics/international-cannabis-consortium-icc/. This GWAS was conducted on samples from the International Cannabis Consortium (ICC) and on the UK Biobank. Summary results used in the analyses excluded 23andMe participants (43,380 cases and 118,702 controls). Cannabis use was assessed from self-reported items that asked participants to indicate whether they have ever used cannabis (yes/no).

Further details about these summary statistics can be obtained from the original GWAS publications.

Polygenic score computation

Quality control of GWAS summary statistics was performed prior to conducting polygenic score analysis following recommended procedures[3] to exclude sex chromosomes, palindromic variants and variants with INFO < 0.8 and MAF < 0.01.

A polygenic score is a single value reflecting an individual's genetic propensity for a given trait. More formally, polygenic scores are calculated by taking weighted sum scores of trait-associated SNPs based on the number of effect alleles (0, 1 or 2 for biallelic SNPs) carried by each genotyped participant. SNP weights are obtained from publicly available summary statistics from large and independent GWAS discovery samples. SNP effects are assumed to contribute additively to genetic liability. The scores can be computed based on a few SNPs (typically selected as those SNPs reaching specific p-value thresholds in GWAS) or millions of SNPs (e.g., genome-wide). For mathematical definitions, see our guide on computing polygenic scores.[22]

PRS-PC[23] is a method to compute polygenic scores that takes the first principal component of polygenic scores computed at several p-value thresholds. This approach avoids inflated type-I error as it does not rely on selecting the most predictive polygenic score based on different p-value thresholds (as is typically done) whilst also resulting in polygenic scores that are more predictive than scores from an arbitrarily chosen p-value threshold. PRS-PC were conducted on polygenic scores calculated using PRSice software[24] at seven thresholds ($p < 1 \times 10^{-5}$, 0.0005, 0.005, 0.05, 0.1, 0.5 and 1) with prior clumping to remove SNPs in linkage disequilibrium ($r2 > 0.10$ within a 500kb window).

Multiple imputation of ADHD trait scores to account for missingness

Multiple imputation was performed to impute missing ADHD scores at age 8 years using the R package *mice*.[25] To mitigate violations of the missing-at-random assumption, we included several auxiliary variables previously identified as being associated with ADHD in MoBa.[26] Auxiliary variables were selected at baseline and from earlier assessments less affected by attrition. Seven auxiliary variables were obtained from registry data, including maternal and paternal age at baseline, child sex, birth weight, birth order, maternal number of cigarettes per day smoked at the start of pregnancy, and the number of previous pregnancies. Thirteen auxiliary variables came from early assessments: Annual income and educational attainment for mothers and fathers assessed at baseline; maternal ADHD symptoms, child motor and language development, child ADHD symptoms, child disruptive behaviour symptoms and child anxiety symptoms from maternal reports when children were aged 3 years; maternal reports of children's ADHD at age 5; and children's reading and arithmetic skills assessed at age 8. Parental and child clinical diagnoses of ADHD were obtained from health care records.

Polygenic scores for ADHD and other traits for parents and children (which were available for the full sample) were also included as auxiliary variables. We included the family members' polygenic scores in the imputation process in line with current recommendations to impute based on all variables used in the analytic model [27-29] An additional advantage of including the polygenic scores for the purposes of imputation is that these scores predicted missingness of ADHD trait scores in MoBa (Supplementary Table 4), which is advantageous when imputing missing data under the assumption of missingness not-at-random.

A hundred imputations were performed and final estimates were summarized over repeated analyses by Rubin's rules.[30]

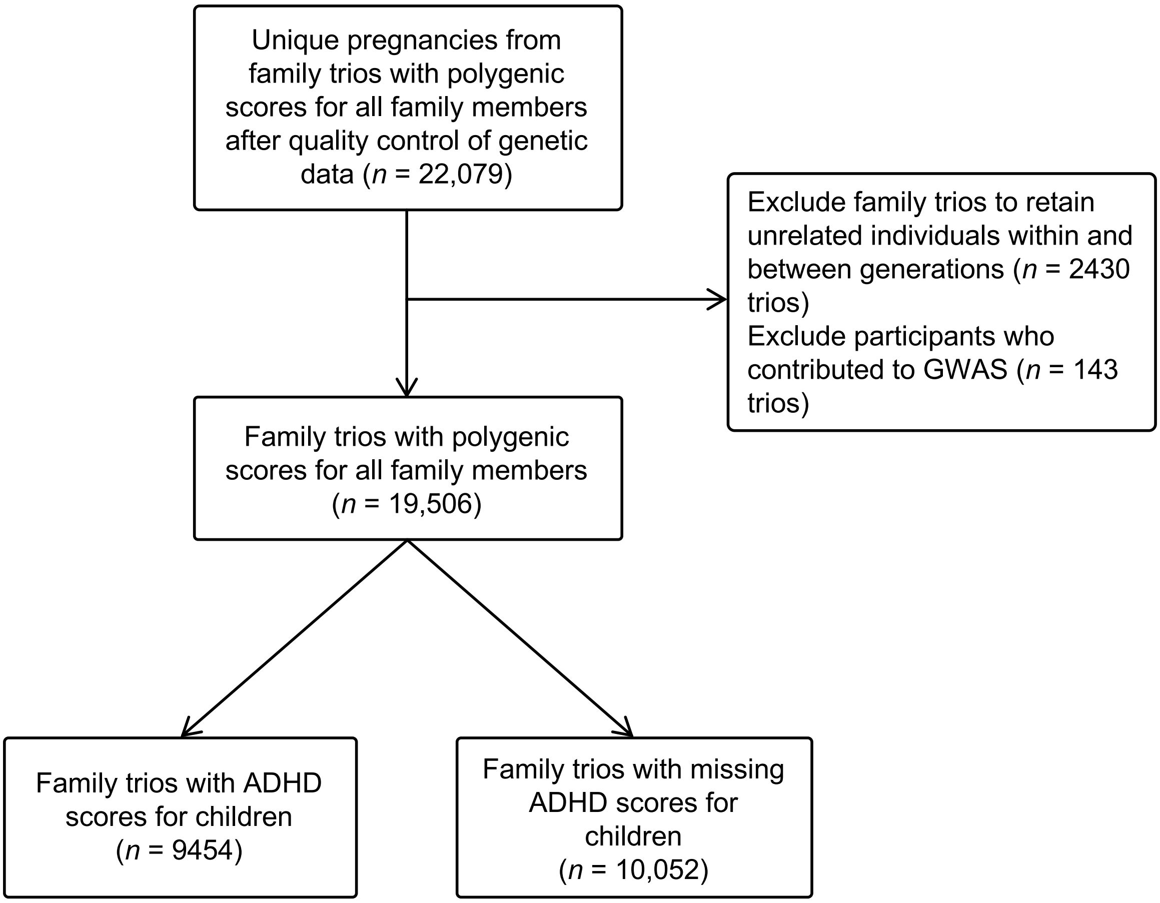

Supplementary Figure 1. STROBE diagram of eligible MoBa sample.

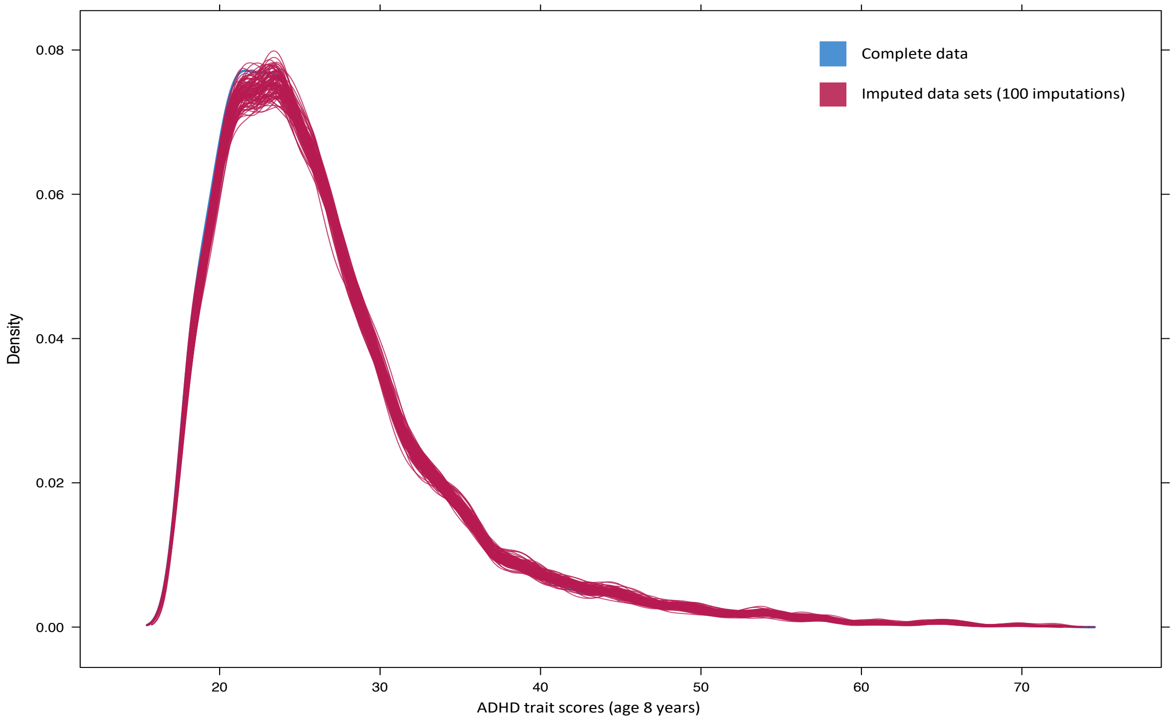

Supplementary Figure 2. Density plot comparing distributions of complete and imputed data.

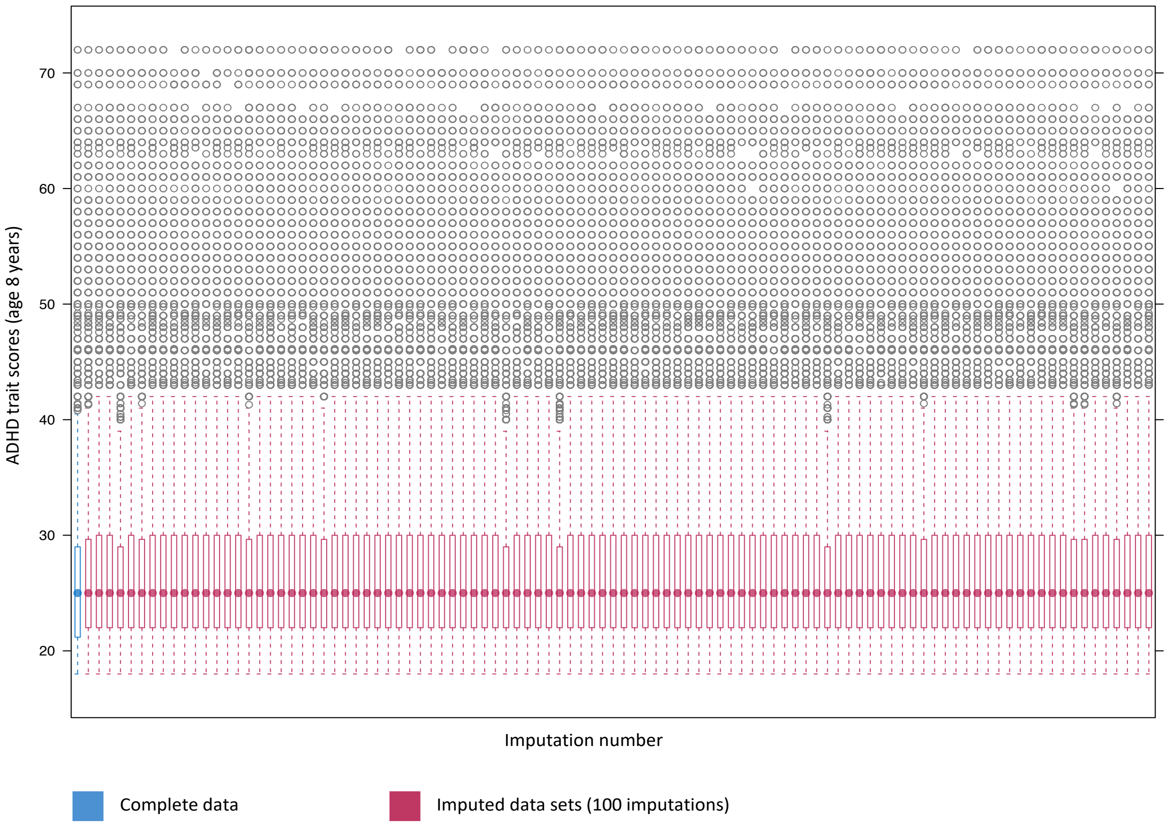

Supplementary Figure 3. Box-and-whisker plot comparing distributions of complete and imputed data.

Supplementary Table 1. Variables included during multiple imputation for missingness of ADHD trait scores.

Variable	N	Mean	Std. Dev.	Min	Max	% Missing	PUC	OS	r_{ADHD}
Imputed variable									
ADHD traits age 8 years	9454	26.54	7.37	18.00	72.00	0.52	0.00	0.00	1.000
Variables in analytic models									
ADHD PGS Child	19506	0.000	0.996	-4.324	4.137	0	1.00	0.52	0.094
ADHD PGS Father	19506	-0.004	1.000	-3.935	4.067	0	1.00	0.52	0.027
ADHD PGS Mother	19506	0.002	1.006	-4.191	4.513	0	1.00	0.52	0.056
Alcohol PGS Child	19506	-0.004	1.000	-3.903	4.095	0	1.00	0.52	0.007
Alcohol PGS Father	19506	-0.007	0.999	-3.941	4.029	0	1.00	0.52	-0.030
Alcohol PGS Mother	19506	0.005	0.996	-3.893	4.097	0	1.00	0.52	0.007
Anxiety PGS Child	19506	0.000	0.997	-3.712	3.904	0	1.00	0.52	0.013
Anxiety PGS Father	19506	0.000	0.998	-4.002	3.878	0	1.00	0.52	0.020
Anxiety PGS Mother	19506	0.006	1.000	-3.905	3.694	0	1.00	0.52	-0.002
ASD PGS Child	19506	-0.001	0.998	-3.967	4.045	0	1.00	0.52	0.038
ASD PGS Father	19506	-0.016	1.003	-3.651	4.158	0	1.00	0.52	0.027
ASD PGS Mother	19506	0.013	1.003	-4.244	4.428	0	1.00	0.52	0.038
Bipolar PGS Child	19506	-0.008	1.004	-3.656	3.688	0	1.00	0.52	-0.022
Bipolar PGS Father	19506	-0.011	0.999	-4.351	3.522	0	1.00	0.52	-0.007
Bipolar PGS Mother	19506	-0.002	1.003	-3.617	4.515	0	1.00	0.52	-0.007
Cannabis PGS Child	19506	-0.006	1.002	-4.225	3.644	0	1.00	0.52	0.000
Cannabis PGS Father	19506	-0.002	0.991	-3.784	3.836	0	1.00	0.52	-0.013
Cannabis PGS Mother	19506	-0.001	0.999	-3.752	4.387	0	1.00	0.52	0.018
Cognition PGS Child	19506	-0.004	1.008	-4.713	3.990	0	1.00	0.52	-0.032
Cognition PGS Father	19506	-0.012	0.995	-3.967	4.205	0	1.00	0.52	-0.012
Cognition PGS Mother	19506	0.002	0.998	-4.061	3.642	0	1.00	0.52	0.003
Depression PGS Child	19506	0.001	0.996	-3.927	4.262	0	1.00	0.52	0.028
Depression PGS Father	19506	0.003	0.997	-3.881	3.778	0	1.00	0.52	0.015
Depression PGS Mother	19506	-0.002	0.999	-3.832	3.721	0	1.00	0.52	0.021
EA PGS Child	19506	-0.007	1.009	-4.167	4.520	0	1.00	0.52	-0.048
EA PGS Father	19506	-0.020	1.006	-3.847	4.023	0	1.00	0.52	-0.029
EA PGS Mother	19506	0.017	0.996	-4.143	3.840	0	1.00	0.52	0.004
Neuroticism PGS Child	19506	0.006	0.998	-3.875	4.250	0	1.00	0.52	0.033
Neuroticism PGS Father	19506	0.004	1.002	-4.459	3.713	0	1.00	0.52	0.019
Neuroticism PGS Mother	19506	-0.006	0.998	-3.903	3.836	0	1.00	0.52	0.043
Schizophrenia PGS Child	19506	-0.002	1.001	-3.894	3.791	0	1.00	0.52	0.005
Schizophrenia PGS Father	19506	-0.011	1.001	-4.142	4.772	0	1.00	0.52	0.005
Schizophrenia PGS Mother	19506	0.006	0.994	-4.094	3.711	0	1.00	0.52	0.005
Smoking PGS Child	19506	-0.007	1.000	-4.169	3.960	0	1.00	0.52	0.054
Smoking PGS Father	19506	0.002	1.001	-3.958	4.138	0	1.00	0.52	0.014
Smoking PGS Mother	19506	0.002	0.997	-3.965	3.816	0	1.00	0.52	0.018
Auxiliary variables									
ADHD traits age 5 years	9134	16.40	4.62	12.00	48.00	0.53	0.25	0.27	0.601
Sex	19506					0	1.00	0.52	-0.170
... Female	9517	48.80%							
... Male	9989	51.20%							
Maternal age	19506	30.09	4.47	17.00	47.00	0	1.00	0.52	-0.059
Paternal age	19506	32.51	5.16	18.00	61.00	0	1.00	0.52	-0.037
Number of previous deliveries	19506	1.73	0.85	1.00	5.00	0	1.00	0.52	-0.079
Maternal CPD (neonatal)	15898	0.71	2.84	0.00	58.00	0.18	0.82	0.52	0.084
Birth weight (grams)	19506	3643.14	511.97	448.00	6300.00	0	1.00	0.52	-0.035
Maternal educational attainment	18221	4.64	1.22	1.00	6.00	0.07	0.92	0.51	-0.079
Paternal educational attainment	17581	4.30	1.41	1.00	6.00	0.10	0.89	0.51	-0.095
Maternal income	18618	4.13	1.34	1.00	7.00	0.05	0.94	0.51	-0.042
Paternal income	18112	5.06	1.33	1.00	7.00	0.07	0.91	0.50	-0.061
Maternal ADHD traits	11711	12.51	3.39	6.00	30.00	0.40	0.39	0.33	0.244

Continued on next page…

Supplementary Table 1. ***(continued)***

Variable	N	Mean	Std. Dev.	Min	Max	% Missing	PUC	OS	r_{ADHD}
Motor delay	11867					0.39	0.39	0.33	0.088
... No	11652	98.20%							
... Yes	215	1.80%							
Language development	11837	5.74	0.55	1.00	6.00	0.39	0.39	0.33	-0.154
ADHD traits age 3 years	11927	9.42	2.25	5.00	18.00	0.39	0.40	0.33	0.382
Anxiety ratings age 3 years	11922	3.70	0.92	2.00	9.00	0.39	0.40	0.33	0.126
Disruptive behaviours age 3 years	11923	7.32	1.62	3.75	15.00	0.39	0.40	0.33	0.306
Reading skills, Grade 1	9194	1.37	0.55	1.00	3.00	0.53	0.00	0.00	0.294
Reading skills, Grade 2	9076	1.31	0.54	1.00	3.00	0.53	0.00	0.00	0.326
Arithmetic skills, Grade 2	9009	1.19	0.44	1.00	3.00	0.54	0.00	0.00	0.252
ADHD diagnosis (child)	19494					0	1.00	0.52	0.453
... No	18579	95.30%							
... Yes	915	4.70%							
ADHD diagnosis (mother)	19494					0	1.00	0.52	0.118
... No	19302	99.00%							
... Yes	192	1.00%							
ADHD diagnosis (father)	19467					0	1.00	0.52	0.105
... No	19291	99.10%							
... Yes	176	0.90%							

Note: % Missing = Percent of missingness in full sample (N = 19,506); PUC = Proportion of usable cases; OS = Outbound statistic; r_{ADHD} = Correlation with ADHD traits (age 8 years); PGS = Polygenic score; ADHD = Attention-deficit/hyperactivity disorder; ASD = Autism spectrum disorder; EA = Educational attainment; CPD = Cigarettes per day.

Supplementary Table 2. Genome-wide association study summary statistics used to compute polygenic scores

Phenotype	Study	*N*	Sample prevalence	Population prevalence	SNP-h^2	*SE*
ADHD	Demontis *et al.* (2019)	53,293	0.358	0.050	0.217	0.014
ASD	Grove *et al.* (2019)	46,351	0.397	0.005	0.096	0.008
Schizophrenia	Pardinas *et al.* (2018)	105,318	0.386	0.010	0.234	0.008
Bipolar disorder	Stahl *et al.* (2019)	51,710	0.396	0.020	0.237	0.012
Depression	Howard *et al.* (2019)	500,199	0.341	0.341	0.100	0.004
Anxiety disorder	Purves *et al.* (2019)	83,566	0.305	0.209	0.165	0.011
Neuroticism	Nagel *et al.* (2018) [a]	390,278	-	-	0.103	0.003
Cognition	Savage *et al.* (2018)	269,867	-	-	0.185	0.008
EA	Lee *et al.* (2018) [b]	765,723	-	-	0.107	0.003
Alcohol use	Linner *et al.* (2019)	414,343	-	-	0.069	0.003
Smoking	Wootton *et al.* (2019)	462,690	-	-	0.094	0.003
Cannabis use	Pasman *et al.* (2018) [a]	162,082	0.268	0.268	0.120	0.008

Note: SNP-h^2 = Single nucleotide polymorphism (SNP) heritability; ADHD = Attention-deficit/hyperactivity disorder; ASD = Autism spectrum disorder; EA = Educational attainment.
[a] excluding 23andMe participants; [b] excluding MoBa and 23andMe participants.

Supplementary Table 3. Correlations between trio's polygenic scores for each risk factor (*N* = 19,506 families).

PGS	Mother-Father	Father-Child	Mother-Child
ADHD	-0.005	0.486	0.494
ASD	-0.013	0.486	0.489
Schizophrenia	0.023	0.502	0.496
Bipolar disorder	0.012	0.496	0.501
Depression	-0.016	0.482	0.489
Anxiety	-0.010	0.496	0.483
Neuroticism	-0.003	0.499	0.489
Cognition	0.041	0.517	0.512
Educational attainment	0.108	0.544	0.536
Alcohol use	0.006	0.481	0.502
Smoking	0.035	0.503	0.507
Cannabis use	0.005	0.492	0.495

Note: PGS = Polygenic score; ADHD = Attention-deficit/hyperactivity disorder; ASD = Autism spectrum disorder.

Supplementary Table 4. Comparison of mean standardized polygenic scores between families with and without child ADHD scores available.

PGS	Role	Mean standardized polygenic scores		*t*	*p*
		Missing ADHD scores (n = 10,052)	Available ADHD scores (n = 9,454)		
ADHD	Child	0.0303	-0.0322	4.38	**1.17×10^{-5}**
	Father	0.0200	-0.0212	2.89	**0.004**
	Mother	0.0397	-0.0420	5.73	**1.01×10^{-8}**
Autism spectrum disorder	Child	-0.0086	0.0091	-1.24	0.215
	Father	-0.0073	0.0077	-1.05	0.292
	Mother	-0.0110	0.0117	-1.59	0.112
Schizophrenia	Child	0.0314	-0.0333	4.53	**5.81×10^{-6}**
	Father	0.0181	-0.0192	2.62	**0.009**
	Mother	0.0530	-0.0561	7.66	**2.00×10^{-14}**
Bipolar disorder	Child	-0.0081	0.0086	-1.17	0.241
	Father	-0.0066	0.0070	-0.96	0.338
	Mother	0.0037	-0.0039	0.53	0.597
Depression	Child	0.0220	-0.0233	3.18	**0.001**
	Father	0.0182	-0.0193	2.63	**0.008**
	Mother	0.0366	-0.0388	5.29	**1.22×10^{-7}**
Anxiety	Child	0.0094	-0.0100	1.36	0.174
	Father	0.0038	-0.0040	0.55	0.581
	Mother	0.0099	-0.0105	1.43	0.154
Neuroticism	Child	0.0202	-0.0214	2.92	**0.004**
	Father	0.0028	-0.0030	0.41	0.681
	Mother	0.0285	-0.0302	4.12	**3.86×10^{-5}**
Cognition	Child	-0.0476	0.0505	-6.89	**5.88×10^{-12}**
	Father	-0.0357	0.0379	-5.16	**2.43×10^{-7}**
	Mother	-0.0474	0.0502	-6.85	**7.54×10^{-12}**
Educational attainment	Child	-0.0566	0.0599	-8.18	**2.94×10^{-16}**
	Father	-0.0387	0.0410	-5.60	**2.21×10^{-8}**
	Mother	-0.0751	0.0795	-10.87	**1.91×10^{-27}**
Alcohol use	Child	0.0006	-0.0006	0.08	0.935
	Father	-0.0009	0.0010	-0.13	0.894
	Mother	0.0106	-0.0112	1.52	0.128
Smoking	Child	0.0464	-0.0491	6.70	**2.09×10^{-11}**
	Father	0.0304	-0.0322	4.39	**1.13×10^{-5}**
	Mother	0.0654	-0.0693	9.46	**3.41×10^{-21}**
Cannabis use	Child	-0.0111	0.0118	-1.61	0.108
	Father	-0.0140	0.0149	-2.03	**0.043**
	Mother	0.0037	-0.0039	0.53	0.596

Note: ADHD = Attention-deficit/hyperactivity disorder.

Supplementary Table 5. Family trios' polygenic scores predicting child ADHD traits before and after adjusting for polygenic scores of other family members (corresponding to Figure 1).

		Unadjusted model results				Trio model results				
PGS	Role	*β*	95% *CI*	*p*	*p*FDR	*β*	95% *CI*	*p*	*p*FDR	*p (Δβ)*
ADHD	Child	0.094	0.077; 0.111	$3.45x10^{-25}$	**$1.24x10^{-23}$**	0.098	0.075; 0.122	$1.11x10^{-15}$	**$4.00x10^{-14}$**	0.573
	Father	0.036	0.02; 0.053	$2.41x10^{-5}$	**$1.45x10^{-4}$**	-0.011	-0.032; 0.009	0.278	0.500	$1.48x10^{-14}$
	Mother	0.051	0.033; 0.068	$3.55x10^{-8}$	**$3.20x10^{-7}$**	0.002	-0.019; 0.023	0.864	0.864	$9.95x10^{-17}$
ASD	Child	0.035	0.017; 0.053	$1.80x10^{-4}$	**0.001**	0.017	-0.008; 0.042	0.187	0.374	0.040
	Father	0.023	0.005; 0.041	0.011	**0.022**	0.015	-0.007; 0.037	0.169	0.359	0.220
	Mother	0.030	0.012; 0.047	0.001	**0.003**	0.022	0; 0.043	0.045	0.148	0.183
Schizophrenia	Child	0.012	-0.005; 0.029	0.172	0.238	0.007	-0.018; 0.032	0.571	0.791	0.585
	Father	0.008	-0.009; 0.026	0.353	0.397	0.005	-0.017; 0.026	0.680	0.816	0.577
	Mother	0.009	-0.009; 0.026	0.320	0.372	0.005	-0.016; 0.027	0.632	0.795	0.567
Bipolar disorder	Child	-0.024	-0.043; -0.006	0.009	**0.020**	-0.031	-0.057; -0.004	0.023	0.084	0.509
	Father	-0.007	-0.025; 0.011	0.421	0.459	0.008	-0.014; 0.03	0.484	0.698	0.019
	Mother	-0.010	-0.028; 0.007	0.255	0.306	0.005	-0.017; 0.027	0.640	0.795	0.025
Depression	Child	0.034	0.016; 0.052	$2.32x10^{-4}$	**0.001**	0.024	-0.001; 0.049	0.059	0.178	0.257
	Father	0.015	-0.003; 0.033	0.097	0.151	0.004	-0.018; 0.026	0.742	0.853	0.088
	Mother	0.029	0.01; 0.047	0.002	**0.007**	0.017	-0.005; 0.039	0.129	0.310	0.051
Anxiety disorder	Child	0.025	0.007; 0.043	0.006	**0.015**	0.022	-0.003; 0.046	0.086	0.221	0.674
	Father	0.016	-0.002; 0.034	0.079	0.129	0.005	-0.016; 0.027	0.621	0.795	0.075
	Mother	0.012	-0.005; 0.03	0.172	0.238	0.002	-0.019; 0.023	0.861	0.864	0.078
Neuroticism	Child	0.038	0.02; 0.056	$3.38x10^{-5}$	**$1.74x10^{-4}$**	0.018	-0.007; 0.044	0.162	0.359	0.030
	Father	0.017	-0.001; 0.034	0.060	0.103	0.008	-0.014; 0.029	0.484	0.698	0.155
	Mother	0.042	0.025; 0.06	$2.56x10^{-6}$	**$1.84x10^{-5}$**	0.033	0.012; 0.055	0.002	**0.011**	0.153
Cognition	Child	-0.027	-0.045; -0.009	0.003	**0.009**	-0.047	-0.072; -0.022	$2.01x10^{-4}$	**0.002**	0.020
	Father	-0.013	-0.031; 0.005	0.170	0.238	0.010	-0.011; 0.032	0.347	0.595	$1.65x10^{-4}$
	Mother	0.005	-0.013; 0.023	0.583	0.600	0.029	0.007; 0.05	0.009	**0.039**	$4.46x10^{-5}$
EA	Child	-0.057	-0.075; -0.039	$6.48x10^{-10}$	**$7.78x10^{-9}$**	-0.081	-0.107; -0.054	$3.82x10^{-9}$	**$6.87x10^{-8}$**	0.018
	Father	-0.036	-0.054; -0.018	$7.45x10^{-5}$	**$3.35x10^{-4}$**	0.003	-0.019; 0.026	0.782	0.853	$1.42x10^{-8}$
	Mother	-0.002	-0.021; 0.017	0.822	0.822	0.041	0.018; 0.064	0.001	**0.004**	$5.01x10^{-10}$
Alcohol use	Child	0.010	-0.007; 0.028	0.243	0.306	0.029	0.006; 0.053	0.015	0.061	0.020
	Father	-0.023	-0.04; -0.005	0.011	**0.022**	-0.037	-0.058; -0.016	0.001	**0.004**	0.017
	Mother	0.012	-0.006; 0.031	0.195	0.261	-0.002	-0.024; 0.02	0.837	0.864	0.019
Smoking	Child	0.058	0.041; 0.076	$5.24x10^{-11}$	**$9.43x10^{-10}$**	0.070	0.045; 0.094	$3.67x10^{-8}$	**$4.40x10^{-7}$**	0.202
	Father	0.021	0.003; 0.038	0.019	**0.034**	-0.014	-0.036; 0.008	0.206	0.389	$3.93x10^{-7}$
	Mother	0.026	0.009; 0.044	0.003	**0.009**	-0.009	-0.029; 0.012	0.417	0.682	$2.12x10^{-10}$
Cannabis use	Child	0.010	-0.007; 0.027	0.254	0.306	0.003	-0.021; 0.027	0.781	0.853	0.447
	Father	-0.006	-0.022; 0.01	0.462	0.489	-0.008	-0.028; 0.012	0.441	0.690	0.767
	Mother	0.023	0.004; 0.041	0.017	**0.032**	0.021	-0.001; 0.043	0.065	0.181	0.796

Note: PGS = Polygenic scores; CI = confidence intervals (unadjusted); pFDR = p-values adjusted for 36 tests at a false-discovery rate (FDR) of 5%; p (Δβ) = p-value for test of difference between betas (Δβ) for bivariate and trio models; ADHD = Attention-deficit/hyperactivity disorder; ASD = Autism spectrum disorder; EA = Educational attainment. ADHD scores were standardized to have a mean of zero and a standard deviation of 1. Trio results adjusted for polygenic scores of other family members. All models adjusted for child sex and year of birth. N = 19,506 family trios.

Supplementary Table 6. Alcohol dependence polygenic scores predicting child ADHD traits.

		Unadjusted model results			Trio model results		
**PGS**	**Role**	***β***	***SE***	***p***	***β***	***SE***	***p***
Alcohol dependence	Child	0.020	0.010	0.050	0.009	0.014	0.256
	Father	0.009	0.010	0.390	0.004	0.012	0.509
	Mother	0.023	0.010	0.025	0.018	0.012	0.519

Note: PGS = Polygenic scores; CI = confidence intervals; Polygenic scores computed based on GWAS for DSM defined alcohol dependence.[31]

Supplementary Table 7. Family trios' polygenic scores predicting child ADHD traits before and after adjusting for polygenic scores of other family members (within risk factors) in sample with complete phenotypic data available (*N* = 9454).

		Unadjusted model results				Trio model results			
PGS	Role	*β*	95% *CI*	*p*	*p*FDR	*β*	95% *CI*	*p*	*p*FDR
ADHD	Child	0.092	0.072; 0.111	1.43×10^{-19}	**5.13×10^{-18}**	0.097	0.07; 0.124	4.21×10^{-12}	**1.52×10^{-10}**
	Father	0.028	0.009; 0.048	0.005	**0.016**	-0.017	-0.041; 0.006	0.150	0.317
	Mother	0.054	0.034; 0.074	8.16×10^{-8}	**1.47×10^{-6}**	0.006	-0.018; 0.03	0.633	0.736
ASD	Child	0.040	0.02; 0.06	8.16×10^{-5}	**4.90×10^{-4}**	0.015	-0.012; 0.042	0.287	0.517
	Father	0.029	0.009; 0.049	0.005	**0.016**	0.022	-0.001; 0.046	0.065	0.181
	Mother	0.036	0.017; 0.056	3.32×10^{-4}	**0.002**	0.030	0.006; 0.054	0.016	0.056
Schizophrenia	Child	0.007	-0.013; 0.027	0.480	0.596	0.004	-0.024; 0.032	0.789	0.851
	Father	0.004	-0.016; 0.024	0.674	0.736	0.002	-0.022; 0.026	0.854	0.878
	Mother	0.006	-0.013; 0.026	0.528	0.633	0.004	-0.02; 0.029	0.715	0.805
Bipolar disorder	Child	-0.022	-0.042; -0.003	0.027	0.064	-0.031	-0.059; -0.003	0.028	0.093
	Father	-0.006	-0.026; 0.014	0.564	0.655	0.009	-0.015; 0.033	0.451	0.647
	Mother	-0.007	-0.027; 0.012	0.463	0.595	0.008	-0.016; 0.032	0.510	0.656
Depression	Child	0.028	0.008; 0.047	0.007	**0.018**	0.022	-0.005; 0.05	0.110	0.247
	Father	0.013	-0.007; 0.033	0.212	0.343	0.002	-0.022; 0.026	0.883	0.883
	Mother	0.020	0; 0.04	0.052	0.112	0.009	-0.015; 0.033	0.466	0.647
Anxiety disorder	Child	0.017	-0.003; 0.036	0.103	0.176	0.013	-0.015; 0.041	0.349	0.546
	Father	0.019	0; 0.039	0.056	0.112	0.013	-0.012; 0.037	0.309	0.530
	Mother	0.000	-0.02; 0.02	0.993	0.993	-0.007	-0.031; 0.017	0.591	0.710
Neuroticism	Child	0.036	0.016; 0.056	4.05×10^{-4}	**0.002**	0.008	-0.02; 0.036	0.568	0.705
	Father	0.019	-0.001; 0.039	0.061	0.116	0.015	-0.009; 0.039	0.222	0.444
	Mother	0.046	0.026; 0.066	5.72×10^{-6}	**4.11×10^{-5}**	0.042	0.018; 0.066	0.001	**0.003**
Cognition	Child	-0.028	-0.048; -0.009	0.005	**0.016**	-0.051	-0.079; -0.023	3.77×10^{-4}	**0.002**
	Father	-0.011	-0.031; 0.009	0.288	0.431	0.014	-0.01; 0.038	0.262	0.497
	Mother	0.005	-0.015; 0.025	0.626	0.704	0.030	0.006; 0.054	0.014	0.056
EA	Child	-0.049	-0.069; -0.029	1.51×10^{-6}	**1.36×10^{-5}**	-0.079	-0.107; -0.05	7.80×10^{-8}	**1.15×10^{-6}**
	Father	-0.026	-0.046; -0.006	0.010	**0.026**	0.012	-0.012; 0.037	0.326	0.534
	Mother	0.004	-0.016; 0.024	0.698	0.739	0.044	0.02; 0.068	3.45×10^{-4}	**0.002**
Alcohol use	Child	0.009	-0.011; 0.028	0.399	0.553	0.034	0.007; 0.062	0.015	0.056
	Father	-0.028	-0.047; -0.008	0.006	**0.018**	-0.044	-0.068; -0.02	2.86×10^{-4}	**0.002**
	Mother	0.008	-0.012; 0.028	0.428	0.571	-0.009	-0.033; 0.016	0.485	0.647
Smoking	Child	0.053	0.033; 0.073	1.45×10^{-7}	**1.75×10^{-6}**	0.076	0.048; 0.104	9.61×10^{-8}	**1.15×10^{-6}**
	Father	0.012	-0.007; 0.032	0.219	0.343	-0.025	-0.049; -0.001	0.041	0.124
	Mother	0.018	-0.002; 0.038	0.075	0.134	-0.020	-0.044; 0.004	0.106	0.247
Cannabis use	Child	0.002	-0.017; 0.022	0.811	0.835	-0.004	-0.031; 0.024	0.803	0.851
	Father	-0.011	-0.03; 0.009	0.299	0.431	-0.009	-0.033; 0.015	0.471	0.647
	Mother	0.019	0; 0.039	0.056	0.112	0.021	-0.003; 0.045	0.084	0.217

Note: PGS = Polygenic scores; CI = confidence intervals (unadjusted); pFDR = p-values adjusted for 36 tests at a false-discovery rate (FDR) of 5%; ADHD = Attention-deficit/hyperactivity disorder; ASD = Autism spectrum disorder; EA = Educational attainment.

Supplementary Table 8. Polygenic scores predicting child ADHD traits adjusted for polygenic scores across risk factors.

PGS	Role	***β***	95% *CI*	*p*	*p (Δβ)*
ADHD	Child	0.082	0.058; 0.106	**$7.18x10^{-11}$**	0.116
	Father	-0.009	-0.03; 0.013	0.426	0.475
	Mother	0.006	-0.016; 0.028	0.596	0.407
Neuroticism	Child	0.000	-0.026; 0.025	0.972	0.077
	Father	0.008	-0.014; 0.029	0.477	0.932
	Mother	0.040	0.018; 0.061	**$3.24x10^{-4}$**	0.215
Cognition	Child	-0.014	-0.041; 0.014	0.320	0.119
	Father	0.013	-0.011; 0.037	0.281	0.696
	Mother	0.019	-0.004; 0.042	0.102	0.255
EA	Child	-0.051	-0.082; -0.02	**0.001**	0.174
	Father	-0.003	-0.028; 0.023	0.843	0.537
	Mother	0.041	0.015; 0.067	**0.002**	0.967
Alcohol use	Child	0.026	0.002; 0.05	**0.031**	0.340
	Father	-0.034	-0.055; -0.013	**0.002**	0.401
	Mother	-0.005	-0.027; 0.018	0.682	0.528
Smoking	Child	0.035	0.009; 0.062	**0.009**	0.099
	Father	-0.009	-0.032; 0.014	0.447	0.448
	Mother	-0.001	-0.023; 0.021	0.932	0.311

Note: PGS = Polygenic scores; CI = confidence intervals (unadjusted); p (Δβ) = p-value for test of difference between betas (Δβ) from within-risk factor models and across risk factor model; ADHD = Attention-deficit/hyperactivity disorder; EA = Educational attainment. ADHD scores were standardized to have a mean of zero and a standard deviation of 1. Model adjusted for child sex and year of birth. N = 19,506 family trios.

Supplementary Table 9. Family trios' polygenic scores predicting child inattention traits before and after adjusting for polygenic scores of other family members (within risk factors).

		Unadjusted model results				Trio model results			
PGS	Role	*β*	95% *CI*	*p*	*p*FDR	*β*	95% *CI*	*p*	*p*FDR
ADHD	Child	0.077	0.059; 0.094	2.22×10^{-16}	**7.99×10^{-15}**	0.088	0.064; 0.112	9.47×10^{-13}	**3.41×10^{-11}**
	Father	0.031	0.012; 0.05	0.001	**0.004**	-0.012	-0.034; 0.01	0.288	0.494
	Mother	0.032	0.015; 0.05	3.32×10^{-4}	**0.002**	-0.011	-0.032; 0.009	0.280	0.494
ASD	Child	0.033	0.014; 0.051	4.79×10^{-4}	**0.002**	0.022	-0.003; 0.047	0.086	0.247
	Father	0.018	0.001; 0.035	0.044	0.083	0.008	-0.014; 0.029	0.488	0.733
	Mother	0.025	0.008; 0.043	0.005	**0.015**	0.015	-0.007; 0.036	0.173	0.384
Schizophrenia	Child	0.014	-0.004; 0.032	0.122	0.183	0.017	-0.008; 0.042	0.192	0.384
	Father	0.005	-0.014; 0.024	0.590	0.609	-0.003	-0.026; 0.02	0.789	0.926
	Mother	0.006	-0.012; 0.024	0.533	0.581	-0.002	-0.025; 0.02	0.833	0.937
Bipolar disorder	Child	-0.023	-0.041; -0.005	0.012	**0.027**	-0.024	-0.048; 0	0.051	0.184
	Father	-0.014	-0.033; 0.006	0.163	0.232	-0.002	-0.025; 0.021	0.870	0.937
	Mother	-0.008	-0.026; 0.01	0.367	0.426	0.004	-0.018; 0.025	0.725	0.926
Depression	Child	0.032	0.014; 0.05	0.001	**0.002**	0.032	0.005; 0.058	0.018	0.073
	Father	0.012	-0.006; 0.03	0.179	0.238	-0.003	-0.026; 0.019	0.783	0.926
	Mother	0.019	0.001; 0.037	0.042	0.083	0.003	-0.019; 0.025	0.791	0.926
Anxiety disorder	Child	0.025	0.007; 0.043	0.006	**0.016**	0.021	-0.004; 0.046	0.106	0.254
	Father	0.017	-0.001; 0.036	0.063	0.113	0.007	-0.016; 0.03	0.542	0.781
	Mother	0.012	-0.007; 0.03	0.215	0.276	0.002	-0.02; 0.024	0.885	0.937
Neuroticism	Child	0.032	0.014; 0.05	4.32×10^{-4}	**0.002**	0.014	-0.011; 0.039	0.276	0.494
	Father	0.014	-0.004; 0.033	0.121	0.183	0.008	-0.014; 0.029	0.486	0.733
	Mother	0.037	0.018; 0.055	1.18×10^{-4}	**0.001**	0.030	0.008; 0.052	0.009	**0.041**
Cognition	Child	-0.027	-0.045; -0.009	0.003	**0.011**	-0.053	-0.078; -0.028	4.48×10^{-5}	**3.23×10^{-4}**
	Father	-0.012	-0.029; 0.005	0.168	0.232	0.014	-0.007; 0.035	0.190	0.384
	Mother	0.010	-0.008; 0.028	0.285	0.342	0.037	0.015; 0.058	0.001	**0.006**
EA	Child	-0.053	-0.071; -0.036	7.60×10^{-9}	**9.12×10^{-8}**	-0.079	-0.105; -0.053	2.82×10^{-9}	**5.08×10^{-8}**
	Father	-0.037	-0.055; -0.019	5.22×10^{-5}	**4.69×10^{-4}**	0.000	-0.021; 0.022	0.967	0.967
	Mother	0.005	-0.013; 0.023	0.592	0.609	0.047	0.025; 0.069	2.76×10^{-5}	**2.48×10^{-4}**
Alcohol use	Child	0.007	-0.011; 0.025	0.424	0.476	0.023	-0.002; 0.047	0.068	0.224
	Father	-0.025	-0.044; -0.007	0.007	**0.018**	-0.036	-0.058; -0.014	0.001	**0.008**
	Mother	0.015	-0.003; 0.033	0.107	0.183	0.004	-0.017; 0.025	0.723	0.926
Smoking	Child	0.053	0.036; 0.071	4.91×10^{-9}	**8.84×10^{-8}**	0.067	0.042; 0.091	1.29×10^{-7}	**1.55×10^{-6}**
	Father	0.025	0.008; 0.042	0.005	**0.014**	-0.008	-0.029; 0.013	0.452	0.733
	Mother	0.015	-0.004; 0.033	0.122	0.183	-0.019	-0.041; 0.003	0.096	0.247
Cannabis use	Child	0.010	-0.008; 0.027	0.282	0.342	0.001	-0.024; 0.027	0.921	0.947
	Father	-0.002	-0.02; 0.016	0.814	0.814	-0.003	-0.025; 0.019	0.797	0.926
	Mother	0.020	0.002; 0.039	0.028	0.060	0.020	-0.003; 0.043	0.093	0.247

Note: PGS = Polygenic scores; CI = confidence intervals (unadjusted); pFDR = p-values adjusted for 36 tests at a false-discovery rate (FDR) of 5%; ADHD = Attention-deficit/hyperactivity disorder; ASD = Autism spectrum disorder; EA = Educational attainment.

Supplementary Table 10. Family trios' polygenic scores predicting child hyperactivity-impulsivity traits before and after adjusting for polygenic scores of other family members (within risk factors).

		Unadjusted model results				Trio model results			
PGS	Role	*β*	95% *CI*	*p*	*p*FDR	*β*	95% *CI*	*p*	*p*FDR
ADHD	Child	0.090	0.071; 0.108	1.39×10^{-19}	**5.02×10^{-18}**	0.089	0.064; 0.114	8.64×10^{-12}	**3.11×10^{-10}**
	Father	0.033	0.014; 0.051	0.001	**0.002**	-0.010	-0.032; 0.012	0.364	0.563
	Mother	0.056	0.039; 0.073	1.81×10^{-10}	**3.25×10^{-9}**	0.012	-0.008; 0.033	0.229	0.457
ASD	Child	0.029	0.011; 0.047	0.001	**0.004**	0.011	-0.014; 0.035	0.391	0.563
	Father	0.022	0.004; 0.039	0.016	**0.042**	0.017	-0.005; 0.038	0.126	0.349
	Mother	0.026	0.007; 0.044	0.007	**0.018**	0.021	-0.002; 0.043	0.070	0.230
Schizophrenia	Child	0.006	-0.011; 0.023	0.484	0.528	-0.004	-0.028; 0.021	0.759	0.795
	Father	0.008	-0.01; 0.026	0.378	0.439	0.010	-0.012; 0.032	0.390	0.563
	Mother	0.008	-0.01; 0.027	0.370	0.439	0.010	-0.013; 0.033	0.380	0.563
Bipolar disorder	Child	-0.019	-0.037; -0.001	0.036	0.076	-0.030	-0.055; -0.004	0.025	0.094
	Father	0.002	-0.016; 0.02	0.845	0.845	0.016	-0.006; 0.039	0.151	0.387
	Mother	-0.010	-0.029; 0.009	0.291	0.381	0.005	-0.018; 0.028	0.694	0.795
Depression	Child	0.029	0.012; 0.047	0.001	**0.003**	0.010	-0.016; 0.036	0.453	0.604
	Father	0.015	-0.004; 0.034	0.123	0.210	0.011	-0.013; 0.034	0.377	0.563
	Mother	0.034	0.016; 0.051	0.000	**0.001**	0.029	0.007; 0.051	0.010	0.073
Anxiety disorder	Child	0.020	0.003; 0.038	0.024	0.058	0.016	-0.008; 0.041	0.193	0.408
	Father	0.011	-0.007; 0.03	0.219	0.319	0.003	-0.019; 0.026	0.773	0.795
	Mother	0.012	-0.006; 0.031	0.186	0.291	0.004	-0.017; 0.026	0.688	0.795
Neuroticism	Child	0.037	0.019; 0.055	6.09×10^{-5}	**3.66×10^{-4}**	0.017	-0.008; 0.042	0.175	0.408
	Father	0.015	-0.002; 0.032	0.081	0.154	0.007	-0.015; 0.028	0.535	0.687
	Mother	0.042	0.024; 0.06	5.22×10^{-6}	**3.76×10^{-5}**	0.034	0.013; 0.055	0.002	**0.015**
Cognition	Child	-0.021	-0.04; -0.002	0.030	0.068	-0.029	-0.055; -0.004	0.026	0.094
	Father	-0.011	-0.029; 0.007	0.222	0.319	0.003	-0.018; 0.025	0.751	0.795
	Mother	-0.002	-0.02; 0.016	0.834	0.845	0.013	-0.009; 0.035	0.246	0.467
EA	Child	-0.050	-0.068; -0.031	1.75×10^{-7}	**1.58×10^{-6}**	-0.065	-0.092; -0.038	2.26×10^{-6}	**4.07×10^{-5}**
	Father	-0.029	-0.046; -0.012	0.001	**0.003**	0.003	-0.018; 0.024	0.768	0.795
	Mother	-0.009	-0.027; 0.009	0.304	0.381	0.025	0.004; 0.047	0.022	0.094
Alcohol use	Child	0.010	-0.007; 0.028	0.249	0.345	0.028	0.004; 0.053	0.025	0.094
	Father	-0.015	-0.033; 0.003	0.109	0.196	-0.029	-0.051; -0.006	0.012	0.073
	Mother	0.006	-0.011; 0.023	0.500	0.529	-0.008	-0.029; 0.013	0.444	0.604
Smoking	Child	0.053	0.036; 0.07	2.81×10^{-9}	**3.37×10^{-8}**	0.061	0.035; 0.086	3.47×10^{-6}	**4.16×10^{-5}**
	Father	0.013	-0.004; 0.03	0.134	0.219	-0.017	-0.039; 0.004	0.107	0.320
	Mother	0.032	0.014; 0.051	0.001	**0.002**	0.002	-0.02; 0.025	0.842	0.842
Cannabis use	Child	0.007	-0.011; 0.025	0.430	0.484	0.006	-0.02; 0.031	0.663	0.795
	Father	-0.009	-0.027; 0.009	0.307	0.381	-0.012	-0.034; 0.01	0.283	0.509
	Mother	0.018	0; 0.035	0.052	0.104	0.015	-0.007; 0.037	0.183	0.408

Note: PGS = Polygenic scores; CI = confidence intervals (unadjusted); pFDR = p-values adjusted for 36 tests at a false-discovery rate (FDR) of 5%; ADHD = Attention-deficit/hyperactivity disorder; ASD = Autism spectrum disorder; EA = Educational attainment.

References

1. Chang CC, Chow CC, Tellier LC, Vattikuti S, Purcell SM, Lee JJ. Second-generation PLINK: rising to the challenge of larger and richer datasets. *GigaScience* 2015; **4**: 7.
2. Manichaikul A, Mychaleckyj JC, Rich SS, Daly K, Sale M, Chen WM. Robust relationship inference in genome-wide association studies. *Bioinformatics* 2010; **26**(22): 2867-73.
3. Choi SW, Mak TS, O'Reilly PF. Tutorial: a guide to performing polygenic risk score analyses. *Nat Protoc* 2020; **15**(9): 2759-72.
4. Watanabe K, Stringer S, Frei O, et al. A global overview of pleiotropy and genetic architecture in complex traits. *Nat Genet* 2019; **51**(9): 1339-48.
5. Demontis D, Walters RK, Martin J, et al. Discovery of the first genome-wide significant risk loci for attention deficit/hyperactivity disorder. *Nat Genet* 2019; **51**(1): 63-75.
6. Neale BM, Medland SE, Ripke S, et al. Meta-analysis of genome-wide association studies of attention-deficit/hyperactivity disorder. *J Am Acad Child Adolesc Psychiatry* 2010; **49**(9): 884-97.
7. Grove J, Ripke S, Als TD, et al. Identification of common genetic risk variants for autism spectrum disorder. *Nat Genet* 2019; **51**(3): 431-44.
8. Cross-Disorder Group of the Psychiatric Genomics Consortium. Identification of risk loci with shared effects on five major psychiatric disorders: a genome-wide analysis. *Lancet* 2013; **381**(9875): 1371-9.
9. Pardinas AF, Holmans P, Pocklington AJ, et al. Common schizophrenia alleles are enriched in mutation-intolerant genes and in regions under strong background selection. *Nat Genet* 2018; **50**(3): 381-9.
10. Stahl EA, Breen G, Forstner AJ, et al. Genome-wide association study identifies 30 loci associated with bipolar disorder. *Nat Genet* 2019; **51**(5): 793-803.
11. Howard DM, Adams MJ, Clarke TK, et al. Genome-wide meta-analysis of depression identifies 102 independent variants and highlights the importance of the prefrontal brain regions. *Nat Neurosci* 2019; **22**(3): 343-52.
12. Purves KL, Coleman JRI, Meier SM, et al. A major role for common genetic variation in anxiety disorders. *Mol Psychiatry* 2019.
13. Nagel M, Jansen PR, Stringer S, et al. Meta-analysis of genome-wide association studies for neuroticism in 449,484 individuals identifies novel genetic loci and pathways. *Nat Genet* 2018; **50**(7): 920-7.
14. Eysenck SB, Eysenck HJ, Barrett P. A revised version of the psychoticism scale. *Pers Individ Dif* 1985; **6**(1): 21-9.
15. Costa P, McCrae R. NEO five-factor inventory (NEO-FFI). *Odessa, FL: Psychological Assessment Resources* 1989; **3**.
16. Savage JE, Jansen PR, Stringer S, et al. Genome-wide association meta-analysis in 269,867 individuals identifies new genetic and functional links to intelligence. *Nat Genet* 2018; **50**(7): 912-9.
17. Lee JJ, Wedow R, Okbay A, et al. Gene discovery and polygenic prediction from a genome-wide association study of educational attainment in 1.1 million individuals. *Nat Genet* 2018; **50**(8): 1112-21.
18. Karlsson Linnér R, Biroli P, Kong E, et al. Genome-wide association analyses of risk tolerance and risky behaviors in over 1 million individuals identify hundreds of loci and shared genetic influences. *Nat Genet* 2019; **51**(2): 245-57.
19. Leffondré K, Abrahamowicz M, Xiao Y, Siemiatycki J. Modelling smoking history using a comprehensive smoking index: application to lung cancer. *Stat Med* 2006; **25**(24): 4132-46.
20. Wootton RE, Richmond RC, Stuijfzand BG, et al. Evidence for causal effects of lifetime smoking on risk for depression and schizophrenia: a Mendelian randomisation study. *Psychol Med* 2019: 1-9.
21. Pasman JA, Verweij KJH, Gerring Z, et al. GWAS of lifetime cannabis use reveals new risk loci, genetic overlap with psychiatric traits, and a causal influence of schizophrenia. *Nat Neurosci* 2018; **21**(9): 1161-70.
22. Allegrini AG, Baldwin JR, Barkhuizen W, Pingault JB. Research Review: A guide to computing and implementing polygenic scores in developmental research. *J Child Psychol Psychiat* 2022.
23. Coombes BJ, Ploner A, Bergen SE, Biernacka JM. A principal component approach to improve association testing with polygenic risk scores. *Genet Epidemiol* 2020; **44**(7): 676-86.
24. Choi SW, O'Reilly PF. PRSice-2: Polygenic Risk Score software for biobank-scale data. *GigaScience* 2019; **8**(7): giz082.
25. van Buuren S, Groothuis-Oudshoorn K. mice: Multivariate Imputation by Chained Equations in R. *2011* 2011; **45**(3): 67.
26. Oerbeck B, Overgaard KR, Pripp AH, Reichborn-Kjennerud T, Aase H, Zeiner P. Early Predictors of ADHD: Evidence from a Prospective Birth Cohort. *J Atten Disord* 2020; **24**(12): 1685-92.
27. Kontopantelis E, White IR, Sperrin M, Buchan I. Outcome-sensitive multiple imputation: a simulation study. *BMC Med Res Methodol* 2017; **17**(1): 2.

28. van Ginkel JR, Linting M, Rippe RCA, van der Voort A. Rebutting Existing Misconceptions About Multiple Imputation as a Method for Handling Missing Data. *J Pers Assess* 2020; **102**(3): 297-308.
29. Van Buuren S. Flexible imputation of missing data: CRC press; 2018.
30. Rubin DB. Multiple imputation for nonresponse in surveys: John Wiley & Sons; 2004.
31. Walters RK, Polimanti R, Johnson EC, et al. Transancestral GWAS of alcohol dependence reveals common genetic underpinnings with psychiatric disorders. *Nat Neurosci* 2018; **21**(12): 1656-69.
